# Small Bowel Evisceration through the Vaginal Vault: A Rare Surgical Emergency

**DOI:** 10.7759/cureus.5947

**Published:** 2019-10-20

**Authors:** Abdullah M Rana, Abdul Ahad Rana, Yasser Salama

**Affiliations:** 1 Colorectal Surgery, Royal North Shore Hospital, Sydney, AUS; 2 Surgery, Royal Adelaide Hospital, Adelaide, AUS

**Keywords:** small bowel evisceration, perineal herniation, transabdominal vaginal repair

## Abstract

Small bowel evisceration through the vagina is a rare surgical emergency that requires urgent surgical intervention because of the risk of developing acute small bowel ischemia. We present a case of a 91 year old female presenting with acute small bowel evisceration with majority of her small bowel visible outside the vagina. The bowel wall was edematous requiring emergent laparotomy and reduction of bowel with repair of the vaginal vault. The patient did not require bowel resection. Transvaginal small bowel evisceration is uncommonly described in the literature. Rare cases are reported in elderly, post-menopausal women who have undergone hysterectomy. Multiple approaches to surgical management including laparoscopic, open abdominal, transvaginal as well as combined approaches have been described. Perineal herniation must be kept in the differential in elderly post-hysterectomy patients with sudden onset of abdominal pain with urgent surgical intervention advised.

## Introduction

Transvaginal small bowel evisceration is a rare but potentially life-threatening emergency requiring urgent surgical intervention to prevent its morbidity and mortality. The risk factors for this presentation include advanced age, enterocele, and previous transvaginal surgery. It usually presents with small bowel obstruction on imaging or diagnostic laparotomy showing a small bowel loop herniating through the vagina [1]. Rarely, it presents dramatically with large loops of small bowel prolapsing through the vagina causing significant abdominal discomfort and acute risk of loss of bowel viability [2]. Our patient presented with sudden onset severe abdominal pain and small bowel evisceration through the vagina requiring emergent surgical intervention.

## Case presentation

We present a case of a 91 year old female with a history of hypertension living independently who presented with sudden onset of severe abdominal pain described as sharp and at an intensity that she had never experienced before. She also reported that she felt she ‘had opened her bowel as the trousers felt heavy’. She had associated light-headedness. The patient’s detailed history showed that she did not have constipation or a coughing episode that would increase the intra-abdominal pressure. She had undergone a laparoscopic hysterectomy and oophorectomy for ovarian cancer a year ago and was on hormonal treatment with the last Computed Tomography (CT) scan showing no evidence of disease.

**Figure 1:**
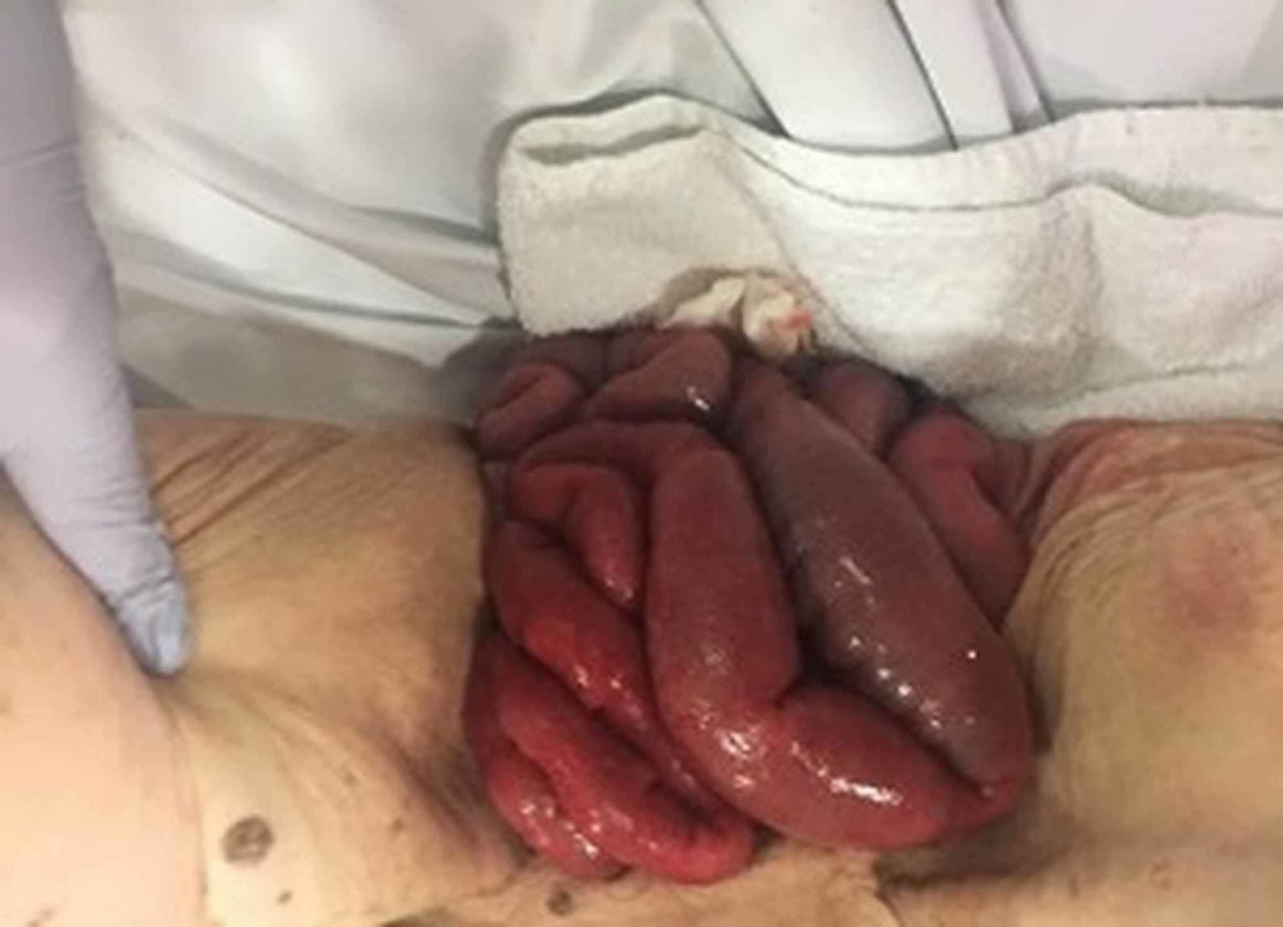
Edematous loops of small bowel hanging outside the vagina with dusky appearing mesentery.

In the emergency room, the patient's blood pressure was 185/75 mmHg, heart rate was 95 per minute sinus rhythm and temperature of 37.8 celsius. She required intravenous analgesia and fluid resuscitation. On examination, her abdomen was distended and tender in the supra-pubic area. Perineal exam showed large prolapse of small bowel with at least 200 cm of bowel visible outside the vagina, the loops appearing edematous and the mesentery dusky and ecchymotic with signs of impaired venous return (figure 1). It was impossible to insert a urinary catheter and do a rectal exam due to the amount of bowel covering the perineal region.

Blood work revealed a pH of 7.48, lactate of 3.5 mg/dL, hemoglobin 11.3 gm/dL and total white cell count 10,500 per microliter. She underwent fluid resuscitation with Hartman’s solution. Small bowel was wrapped with saline soaked packs. Urgent anesthesia assessment was undertaken and she was taken to the operating room.

A mid-line laparotomy was performed and she was noted to have a distended bladder reaching above the umbilicus. The small bowel loops were reduced, the bladder spontaneously decompressed, an indwelling catheter was inserted and the patient was placed in lithotomy position. On examination of the pelvis, a defect was seen in the post-vaginal fornix. The recto-vaginal septum was dissected and the vagina mobilized posteriorly and anteriorly; the plane between the bladder and the vagina was mobilized, and defect edges were debrided. A transabdominal repair was undertaken with closure in layers with Prolene sutures. The peritoneum from the lateral pelvic side walls was mobilized and the repair covered. The peritoneal cavity was washed with six liters of normal saline as the small bowel was eviscerated for at least four hours.

The patient recovered well post-operatively and had return of bowel function within three days of surgery with discharge from the hospital on post-operative day five.

## Discussion

Transvaginal bowel evisceration is a rare surgical presentation first described by Hypernaux *et al* in 1864. There are only about 100 cases reported in the literature and according to the scant data, about 70% of the patients are post-menopausal women [3, 4]. Of those, 73% had previous vaginal surgery and 63% had enterocele. This increased incidence in post-menopausal women has been associated with decreased vaginal wall vascularity and atrophy of the vaginal wall. In pre-menopausal women, it is associated with sexual activity and vaginal trauma.

The risk factors in older age women are vaginal surgery, enterocele repair, increased intra-abdominal pressure with ascites, constipation and increased bouts of coughing. Other risk factors include perineal proctectomy and previous pelvic radiotherapy [5]. After radiotherapy, changes of progressive obliterative endarteritis are noted which result in hypoxia and hypocellularity in the tissue. Eventually there is cell and tissue loss. This usually occurs with high total radiation dose or as a result of direct trauma. Also at higher risk are patients with gynecologic malignancies because of vaginal cuff dehiscence [2]. 

Patients usually present with pelvic or vaginal pain, vaginal bleeding, and the sensation of a mass within the vaginal vault or between the legs. The terminal ileum is most commonly the protruding viscus, although other organs, such as the omentum, salpinx, and epiploic appendices, have also been described [1].

Transvaginal small bowel evisceration is a surgical emergency and is associated with 6-8% risk of mortality. Urgent reduction of small bowel should be undertaken and in rare cases, if the small bowel is not compromised the repair can be transvaginal [6]. The complication has a high morbidity of 15% and 20% of patients may require a bowel resection.

On presentation, the bowel must be thoroughly examined and viability assessed [2]. A reduction may be attempted, however, in most cases such as ours, there is threatened bowel unable to be reduced, thus requiring laparotomy and transabdominal vaginal repair. In the reported literature, the transvaginal and transabdominal surgical approaches have been used. If the bowel is easily reducible, has not suffered prior radiation injury and there is no evidence of an acute abdomen, a vaginal approach may be feasible. On comparison, both the vaginal and abdominal approaches have equal rates of recurrence and complications. Hence, either approach may be utilized depending in the patient presentation [5].

## Conclusions

Early detection and surgical management of this rare surgical emergency is imperative to prevent small bowel ischemia requiring resection and development of sepsis and systemic inflammatory response syndrome (SIRS) because of bowel necrosis which can eventually lead to death.
